# Psychophysiological Responses in Virtual Reality for Assessing Current Nicotine Use and Future Addiction Risk Among Young Adults: Protocol for a Mixed Methods Study

**DOI:** 10.2196/89382

**Published:** 2026-04-24

**Authors:** Shu Wei, Shawn Van, Chris Shia, Michael Gancz, Deepa Camenga, Kammarauche Aneni, Asher Marks, Kimberly Hieftje

**Affiliations:** 1 Department of Pediatrics, Yale School of Medicine, PO Box 208064, New Haven, CT, 06511, United States, +1 (347) 931-0195; 2 Yale College, New Haven, CT, United States; 3 Stanford Center for Computer Research in Music and Acoustics (CCRMA), Stanford University, Stanford, CA, United States; 4 Department of Emergency Medicine, Yale School of Medicine, New Haven, CT, United States; 5 The Child Study Center, Yale School of Medicine, New Haven, CT, United States; 6 Biomedical Informatics and Data Science, Yale School of Medicine, New Haven, CT, United States

**Keywords:** virtual reality, physiological data, nicotine addiction, vaping, eye tracking

## Abstract

**Background:**

Nicotine addiction among youth is a continuing public health concern, and vaping serves as a major pathway to nicotine use. Conventional assessments of craving and addiction risk rely on self-reports, which are prone to bias and lack sensitivity to real-time processes. Virtual reality (VR) enables controlled cue exposure while capturing real-time multimodal data, including subjective experiences, behavioral patterns, and physiological responses, which offer a more implicit and dynamic approach to identifying addiction risk.

**Objective:**

This pilot study examines whether subjective craving and psychophysiological responses (eg, eye gaze, heart rate, and electrodermal activity) to vaping-related cues in VR can distinguish young adults who vape from those who do not. Our secondary objective is to explore associations between these multimodal biomarkers and self-reported measures of craving, dependence, motivation to quit, and susceptibility to initiate vaping.

**Methods:**

Bespoke VR scenes were developed with input from a youth advisory board to ensure ecological validity. Forty young adults aged 18 to 21 years (20 vapers and 20 nonvapers) will complete a single laboratory session. Participants will experience 3 VR scenes (neutral baseline, vaping cues without social pressure, and vaping cues with social pressure in counterbalanced order). Eye gaze, heart rate, and electrodermal activity will be recorded continuously. Participants will complete standardized assessments of craving, sense of presence, and social presence in VR after each cue scene, followed by a short interview at the end. Quantitative data will be analyzed using mixed-model ANOVAs, correlation metrics, and exploratory regularized regression analyses to examine relationships between physiological responses, behavioral measures, and vaping status.

**Results:**

The project received institutional review board approval in August 2025 and was registered publicly in the Open Science Framework in November 2025. Development of the VR stimuli was completed in December 2025. Participant recruitment and data collection began in February 2026, and 7 participants have been enrolled as of March 2026.

**Conclusions:**

This protocol outlines a pilot study integrating immersive VR and multimodal biometrics to examine vaping cue reactivity in young adults. The findings will guide the development and evaluation of VR-based psychophysiological tools for identifying early markers of nicotine use risk. This work will also lay the foundation for adapting the approach to younger adolescents to support scalable early detection and prevention of nicotine addiction and initiation.

## Introduction

### Background

Nicotine addiction is a critical public health concern, particularly among youth who are vulnerable to initiation through electronic nicotine delivery systems (ie, vaping or e-cigarettes) [1]. Early exposure to nicotine during adolescence can disrupt neurodevelopment, heighten impulsivity, and increase the risk of long-term dependence [2,3]. Due to the ongoing development of their brain’s reward system, young people are especially sensitive to reward-based learning, making them more susceptible to adopting and maintaining addictive behaviors such as vaping. Vaping exposes individuals to aerosols containing toxic particles and chemicals that impair lung function and trigger inflammation [4]. It also increases the likelihood of transitioning to conventional cigarette smoking, compounding long-term health risks such as cardiovascular disease, chronic respiratory illness, and cancer [5]. Given the continued high rates of vaping among young adults [6,7], there is an urgent need for effective early prevention and detection strategies to mitigate nicotine addiction.

Virtual reality (VR) has emerged as a promising tool to study and treat nicotine addiction [8-13]. It enables the creation of realistic cue environments that replicate real-world triggers for nicotine craving and use under controlled yet ecologically valid conditions. For instance, Bordnick et al [13] assessed people’s cravings when exposed to virtual cigarette smoking–related cues to understand their smoking behaviors. A growing body of work has further explored how to best optimize the design of VR scenarios to maximize craving induction, such as manipulating visual context (eg, adding more nicotine product–related images) [14], sensory integration (eg, introducing olfactory cues) [15], or social pressure components [16-18]. Building on these cue exposure paradigms, research has also explored how VR can support prevention and cessation efforts. Pericot-Valverde et al [12], for instance, applied a paradigm to help participants practice cigarette refusal skills. Additionally, Wiser et al [10] and Borelli et al [19] have demonstrated that interactive VR games can enhance engagement and support vaping prevention and cessation among youth. By leveraging VR-enabled features such as immersive learning, tailored content design, and simulated skills practice, these applications suggest that VR could effectively supplement existing treatment and prevention modalities for nicotine addiction.

A key advantage of VR, as compared to other interactive modalities for assessing substance use risk [20], is its capacity for synchronized tracking of behavioral and physiological responses during immersive cue exposure. Existing research has shown that nicotine-related cues, whether physical (eg, cigarette packaging) or virtual (eg, images or videos), can elicit stronger emotional and behavioral responses in cigarette smokers than in nonsmokers [21,22]. Metrics such as attentional bias (measured via eye tracking) and autonomic arousal (eg, heart rate [HR] and electrodermal activity [EDA]) capture implicit and automatic processes that may reveal cue reactivity, substance use tendencies, and motivation to consume nicotine [23]. These biometric responses may address important limitations of existing measures of risk, as most rely on self-report, are prone to social desirability and recall bias, and have limited sensitivity to real-time emotional and cognitive processes underlying addiction [8,24]. Furthermore, VR-elicited biometrics may provide an alternative to biological specimens by serving as noninvasive biomarkers for vaping and nicotine use [25]. Together, VR-based psychophysiological methods may serve as valid and acceptable objective tools for identifying nicotine use and predicting addiction risk.

However, despite the use of VR to study cigarette smoking cue reactivity, little work has examined whether VR can distinguish current and future nicotine vaping behaviors. To date, studies have largely used cigarette smoking cue environments to differentiate individuals based on psychophysiological responses using single modalities such as electroencephalogram and eye-tracking [17,26]. Furthermore, no current work has combined multimodal physiological data (eg, visual attention and physiological arousal) with psychological measures (eg, impulsivity, craving, and motivation to quit) to model individuals’ intentions to initiate or cease vaping. Such predictive modeling could play a crucial and timely role in identifying at-risk individuals early and tailoring interventions accordingly.

This study addresses these critical gaps by evaluating whether psychophysiological responses to vaping cues in VR can differentiate nicotine vaping status among young adults. It also explores the associations between biometric reactivity, nicotine vaping status and intention, and individuals’ addiction-related traits (eg, impulsivity and emotion regulation). The long-term goal is to inform the development of real-time adaptive systems capable of detecting and predicting vaping-related intentions, addiction, and initiation risk in naturalistic VR settings, and delivering tailored behavioral-change interventions. This will support the development of early, personalized prevention strategies to enhance health outcomes among youth.

### Aims

#### Overview

The overall goal of this study is to evaluate the feasibility of VR-based psychophysiological assessment methods as an early detection and risk prediction tool for nicotine addiction. This paper presents a mixed methods experimental study protocol designed to examine how psychophysiological responses to vaping cues in VR differ among young adults based on their nicotine vaping status and intentions. The study integrates psychological, physiological, and qualitative measures across 2 bespoke VR cue exposure scenes. Participants include two groups: (1) young adults who currently vape (vapers), for assessment of nicotine dependence and quit intentions; and (2) individuals who have never vaped (nonvapers), for evaluation of susceptibility to initiation.

#### Primary Aim

The primary aim is to examine differences in psychological (craving, sense of presence, and social presence) and physiological (eye gaze, HR, and EDA) responses between young adults who currently vape and those who have never vaped during exposure to VR vaping cues presented with and without social pressure. We hypothesize that vapers will exhibit stronger craving, a greater sense of presence and social presence, increased visual attention to vaping cues, and heightened physiological arousal compared with nonvapers across both VR scenes. We further expect that both groups will show stronger psychological and physiological responses in the social pressure condition than in the no pressure condition.

#### Secondary Aim

The secondary aim is to assess correlations between multimodal physiological measures (eye tracking, HR, and EDA) and subjective vaping-related outcomes, including craving, sense of presence, social presence, nicotine dependence, and quitting intention (among vapers), as well as susceptibility to initiate vaping (among nonvapers). We hypothesize that psychophysiological responses during VR cue exposure will be significantly associated with subjective vaping-related measures, such that greater attentional and autonomic reactivity will correlate with higher craving, dependence, and quit intention among vapers, and with higher susceptibility to initiate vaping among nonvapers. As an exploratory analysis, we will further examine whether combinations of physiological and subjective measures predict vaping status (vaper vs nonvaper).

#### Qualitative Aim

Following the experimental section, participants will complete a brief semistructured interview to understand their perceptions of the VR scenes, their awareness of vaping cues, and their cognitive, affective, and physiological responses to the cues. These qualitative insights will contextualize the study’s quantitative findings and inform the feasibility and acceptability of the VR scenarios for vaping behavior assessment and intervention.

## Methods

### Study Design

This study will examine individuals’ psychological, behavioral, and physiological responses to e-cigarette cues in VR among young adults aged 18 to 21 years. We focus on this age range because the transition into early adulthood often brings increased exposure to vaping-related social contexts (eg, college gatherings) [27], and this pilot allows us to first test the paradigm in adults before adapting it for adolescent populations. A between-subjects design is used to compare current e-cigarette users and nonusers, with a within-subject, counterbalanced component to assess 2 cue conditions (with and without social pressure). This mixed-design approach enables testing of cue effects and group differences while controlling for individual variability.

Prior VR nicotine cue reactivity studies have reported medium-to-large effects for the response difference between user and nonuser groups [17,28]. Based on the relevant research and using G*Power 3.1 (Heinrich Heine University Düsseldorf) for a 2 (group: vapers vs nonvapers)×2 (cue condition: social pressure vs no social pressure) mixed design, we powered the study for the group × cue condition interaction (group×cue condition) assuming a medium effect (*f*=0.25; partial η-squared ≈ 0.06; power=0.80). We assumed a within-participant correlation of *r*=0.50 between cue conditions and a nonsphericity correction of *ε*=1.0 (sphericity is not applicable with 2 within-subject levels). This yields a minimum required sample of 34 (n=17 per group). To ensure robustness and account for potential data loss (eg, eye-tracking dropout and physiological artifacts), we plan to recruit 40 participants in total (n=20 vapers and n=20 nonvapers).

### Study Population and Study Flow

This study will recruit young adults aged 18 to 21 years living in or around New Haven, Connecticut. Recruitment efforts will focus on two distinct groups: (1) individuals who currently use nicotine-containing e-cigarettes, defined as use on 10 days or more within the past 30 days (*vapers*), and (2) individuals who report never having used nicotine-containing vapes (*nonvapers*). Participants who use nicotine in non–e-cigarette forms (eg, cigarettes and nicotine pouches) will not be excluded but will be assessed at baseline so that such exposure can be accounted for analytically. This design allows the comparison of psychophysiological cue reactivity between vapers and nonvapers, as well as the exploration of predictors of future initiation risk among nonvapers.

Eligibility will require participants to be fluent in English and able to provide informed consent. Exclusion criteria include self-reported photosensitive epilepsy, significant visual, hearing, or mobility impairment, current or recent (past 30 days) use of cannabis-containing e-cigarettes, current use of other illicit substances (eg, 3,4-methylenedioxymethamphetamine or ecstasy, and methamphetamine), nonmedical misuse of prescription drugs, or reliance on eyeglasses that are incompatible with VR eye-tracking hardware. These exclusions help reduce noise in psychophysiological responses that may arise from nonnicotine substances, ensuring clearer differentiation between the study groups. A total sample of 40 participants will be enrolled, which is sufficient for detecting medium group effects in VR cue-reactivity studies while accounting for potential data loss.

**Figure 1.**
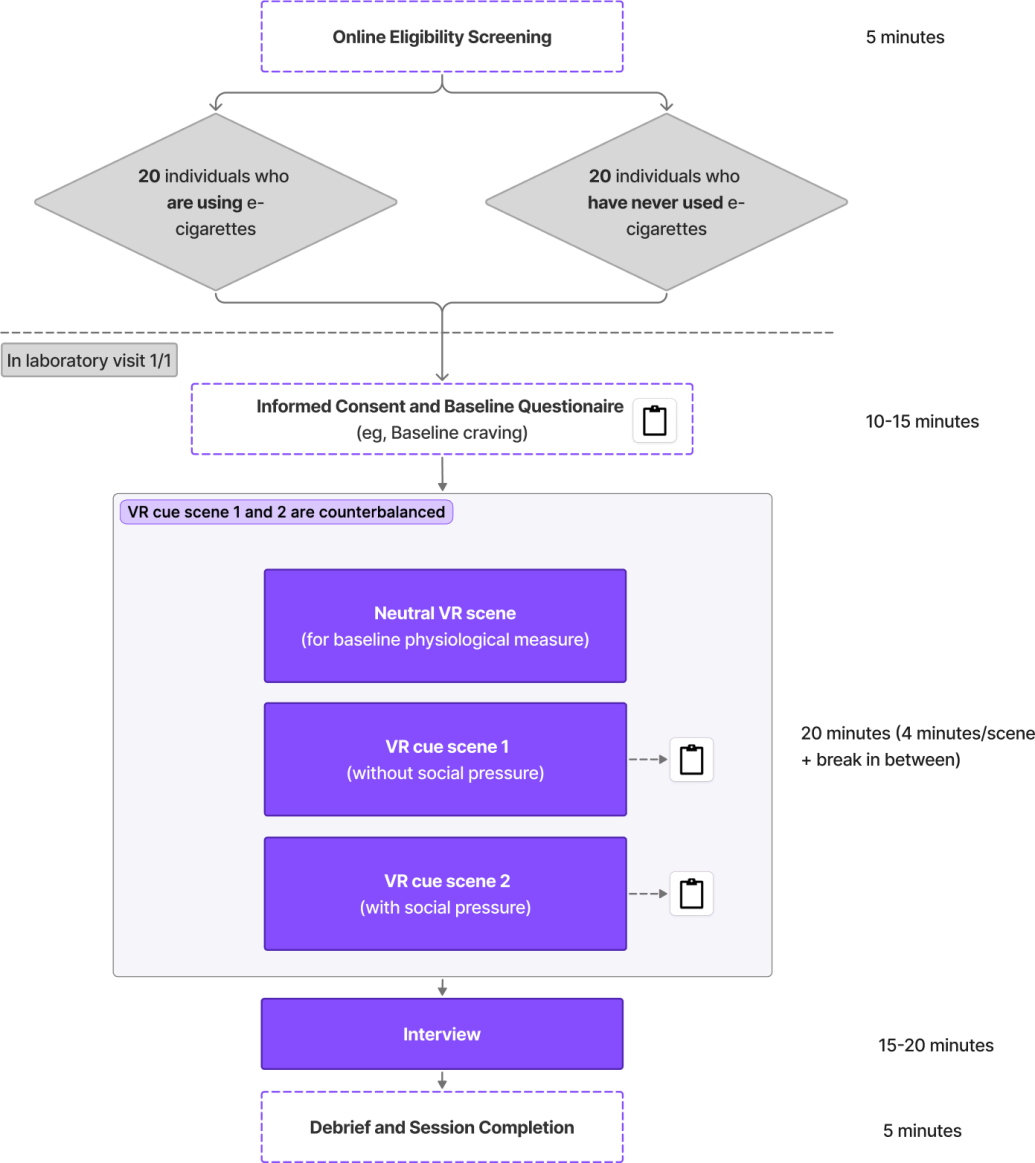
Study flow graph. VR: virtual reality.

The study flow is shown in Figure 1. Eligible participants will complete a single laboratory session lasting approximately 1 hour. After providing informed consent and completing baseline questionnaires, participants will be fitted with a VR headset and biometric sensors. They will complete 3 VR scenes: a neutral baseline, a vaping cue scene without social pressure, and a vaping cue scene with social pressure in a counterbalanced order. Physiological data will be recorded continuously, and brief self-report measures (craving, presence, and social presence) will follow each cue scene. Participants will rate the system’s usability after finishing all the VR sessions. The session will conclude with a short semistructured interview, debriefing, and compensation.

### Stimuli Design and Development

#### User-Centered Stimuli Design

##### Overview

We adopted a user-centered design (UCD) approach to ensure that the VR cue stimuli were both realistic and engaging for young adults [29]. Two focus groups (n=7; 6 females and 1 male; aged 18‐21 y) were conducted. Focus group participants were asked to indicate, on a scale of 1 (“least frequent”) to 5 (“most frequent”), the frequency with which they were in environments where vaping was present (mean 1.9, SD 1.39).

The youth advisory board provided input on settings, character design, and social dynamics relevant to vaping. Each session lasted approximately 90 minutes and included: (1) a warm-up ice-breaking activity, (2) improvisational conversation exercises, (3) co-design of dialogue based on prompt lines, and (4) open feedback and discussion. To protect privacy and encourage creative engagement, participants used pseudonyms throughout the entire session. Advisory board members contributed particularly to dialogue development for the social cue scene, which was designed as a backyard barbecue party.

Rapid qualitative methods [30] were used, wherein 2 research team members took notes during the sessions while recording the audio using a Yale-managed instance of Zoom (Zoom Communications, Inc). Immediately after the discussions, the team reviewed audio recordings, revised field notes, and identified key themes. Across sessions, feedback emphasized the importance of situating vaping cues in familiar, everyday social environments and including both solitary and peer-influenced contexts to capture variability in craving and social pressure.

**Figure 2.**
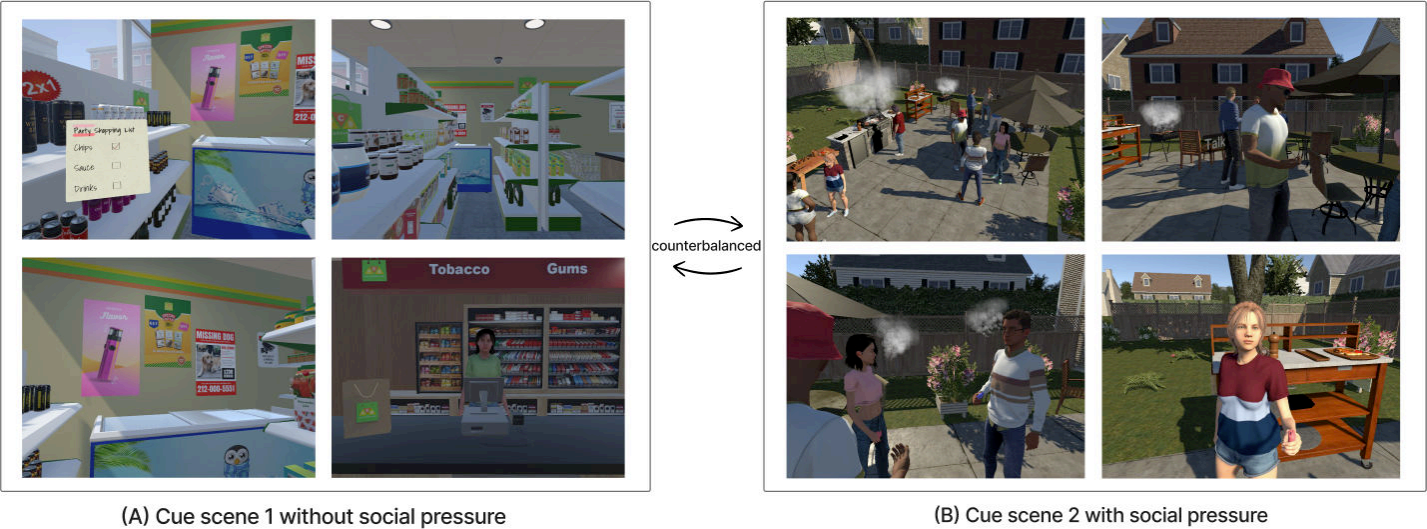
Design of virtual reality vaping cue scenes. (A) No social pressure: participants navigate a convenience store with subtle vaping cues (eg, posters, products). (B) Social pressure: participants attend a barbecue where peers vape and offer an e-cigarette.

Informed by both expert input grounded in existing literature and UCD, 2 bespoke vaping-related cue scenes (Figure 2) were designed and developed for room-scale VR: a convenience store scene without social pressure and a barbecue party scene with social pressure. Each scene lasts approximately 4 minutes and was optimized for immersion, naturalistic interaction, and ecological validity. The “without social pressure” scene follows prior nicotine cue-reactivity paradigms that contrast socially enriched versus socially neutral contexts to isolate interpersonal influence [31,32]. The convenience store setting reflects a common environment where vaping may occur without peer prompting. While this condition minimizes explicit social influence, contextual differences may still affect responses; therefore, the primary contrast is interpreted as the presence versus absence of peer-driven social pressure rather than a fully context-matched manipulation.

##### Cue Scene Without Social Pressure

Participants are placed in a virtual convenience store where they are tasked with picking up items from a shopping list (eg, chips, sauces, and drinks) distributed across areas of the store. This task is designed to enhance ecological validity and introduce a mild cognitive distraction, allowing vaping cues to attract participants’ visual attention more naturally. Cue elements include vaping-related posters, vapes behind the counter, and individuals vaping outside the store, visible through the glass window.

##### Cue Scene With Social Pressure

Participants are immersed in a barbecue party with animated virtual humans, who function both as interaction partners and as background social groups within the scene [33]. Participants are prompted to engage with different clusters of characters and overhear conversations about food, vaping, and school stress. Several characters visibly vape and directly offer an e-cigarette to the participant. This scenario incorporates both visual and verbal peer pressure, reflecting common real-world contexts in which adolescents and young adults may be exposed to vaping.

### Software Development and Apparatus

The VR experience was developed in Unity 2021.3.15 (Unity Technologies) and run on a Windows 11 workstation (Intel i9-14900KF CPU, NVIDIA GeForce RTX 5080 GPU, 64 GB DDR5 RAM). The Meta Quest Pro (Meta Platforms Inc) will be used to deliver the eye-tracking–enabled VR scenes. Eye-gaze behavior will be captured via the headset’s integrated tracker using Oculus Movement software development kit v1.3.2. A set of regions of interest is predefined to register fixations on key stimuli (eg, vaping products, posters, and virtual characters). The system is optimized to minimize false negatives in fixation detection, accounting for the Quest Pro’s accuracy (mean 1.652°, SD 0.699°) when designing the room-scale experience [23,34].

Other physiological data, including EDA and HR, will be recorded using the Shimmer3 galvanic skin response + sensor (Shimmer Sensing) [35], a noninvasive, wrist-worn device placed on the participant’s nondominant hand. The device, worn on the nondominant wrist, measures EDA via galvanic skin response and estimates HR via a photoplethysmography pulse probe. Both signals are processed through validated internal functions within the Shimmer application programming interface to extract features such as skin conductance response (SCR) amplitude and HR metrics. This device is widely used in physiological and participatory research due to its portability and signal accuracy [36-38].

Virtual characters are designed in Character Creator 4 and animated in iClone 8 (Reallusion Inc) using LiveFace for facial motion capture combined with keyframe adjustments, ensuring naturalistic social interactions and emotional expressiveness within the VR environments.

### Recruitment

Participants will be recruited through a combination of physical flyers and digital advertisements disseminated via social media, group chats, and email lists. Flyers will be posted around the New Haven community and college campuses where young adults commonly gather. This multipronged approach will support the recruitment of individuals aged 18 to 21 years living near the study site who can travel for an in-person laboratory visit [39].

To recruit vapers, we will additionally use a snowball recruitment strategy. Because vaping commonly occurs in social settings (eg, college parties) [27], we will contact relevant student organizations to request the distribution of study information to their members. We will also reach out to local vaping retailers and convenience stores to request that they display study materials. Finally, we will reach out to research teams that conducted previous substance use research in and around the New Haven area to request assistance with sharing our recruitment flyer through their mailing lists.

Interested individuals will be directed to a Qualtrics screening questionnaire to determine eligibility. To ensure adequate representation of both vapers and nonvapers, enrollment will be monitored on a weekly basis and adjusted as needed.

### Assessments

At the start of the laboratory visit, participants will complete the informed consent procedure and then part 1 of the assessment questionnaire. Part 1 contains self-report measures on vape perception, substance use intentions and behaviors, and psychosocial assessments (Table 1).

**Table 1.** Baseline and trait-level measures assessing vaping-related behaviors, substance use, and psychosocial characteristics among participants.

Measures	Brief description of items	Response options
For vapers		
Intention to Quit E-cigarettes by *Motivation to Stop Scale (MTSS)* [40]	1 item: “Which of the following describes your intention regarding e-cigarette use?”	7 response options, ranging from (1) “I don’t want to stop using e-cigarettes” to (7) “I REALLY want to stop using e-cigarettes and intend to in the next month”
E-cigarette Dependence by *Penn State Electronic Cigarette Dependence Index measures* [41]	6 items (*α*=.51). An example is: “How many times per day do you usually use your electronic cigarette?”	7 response options, ranging from (1) “0‐4 times/day” to (7) “30+ times/day”
For nonvapers		
Future Addiction by adapted *Pierce Susceptibility Questionnaire* [42]	3 items. An example is: “If one of your best friends offered you an e-cigarette, would you smoke it?”	4-point Likert scale, ranging from (1) “Definitely yes” to (4) “Definitely not”
For all participants		
E-cigarette Perceived Harm and Addiction [43]	3 items. An example is: “How likely is someone to become addicted to e-cigarettes/vaping?”	4-point Likert scale, ranging from (1) “Unlikely” to (4) “Likely”
Other Substance Use (cigarettes, marijuana, alcohol) [44]	6 items. An example is: “How many days in the past 30 days have you smoked a cigarette?”	7 response options, ranging from (1) “0 day” to (7) “All 30 days”
Impulse behavior by *Short UPPS-P Impulsive Behavior Scale* [45]	20 items (*α*=0.74‐0.88), based on the following subscales: Negative urgency (4 items), Lack of perseverance (4 items), Lack of premeditation (4 items), Sensation seeking (4 items), Positive urgency (4 items)	4-point Likert scale, ranging from (1) “Strongly agree” to (4) “Strongly disagree.”
Emotional regulation by *Brief Difficulties in Emotion Regulation Scale (DERS-18)* [46]	18 items (*α*=0.77). An example is: “I pay attention to how I feel.”	5-point Likert scale, ranging from (1) “Almost Never” to (5) “Almost Always”

After each VR cue scene, participants will remove the headset and complete part 2 of the assessment questionnaire. Part 2 contains self-report measures on craving, presence, social presence, and system usability. An overview of the assessment used in the laboratory visit is listed in Table 2.

**Table 2.** Post–VR cue exposure measures assessing craving, presence, social presence, and system usability.

Measures	Items	Response options
Measured twice, after each VR cue scene		
Craving for vaping or smoking e-cigarettes Visual Analog Scale (VAS) [47]	1 item: “I have a desire for vaping/having an e-cigarette right now.”	A visual scale, ranging from 0 (“Not at all”) to 100 (“Extremely”)
Adapted presence and social presence questionnaire from *Presence (IPQ)* and *Social Presence by Networked Minds Measure of Social Presence* [48,49]	1 item from IPQ measuring general presence: “In the computer-generated world I had a sense of ‘being there’” (general presence item from IPQ). 6 items from the co-presence subscale (*α*=0.84). An example is: “The virtual humans caught my attention.”	5-point Likert scale, ranging from (0) “Do not agree at all” to (4) “Very Likely”
Measured once, after all VR scenes		
System usability by V*irtual Reality System Usability Questionnaire (VRSUQ)* [50]	9 items (*α*=0.679‐0.896). An example is: “The system responded well to my manipulations as expected with no delays.”	5-point Likert scale, ranging from (1) “Strongly disagree” to (5) “Strongly agree”

Eye gaze and physiological data will be recorded continuously during both VR cue scenes as follows:

- Eye-gaze behavior: raw eye-tracking data and gaze fixations on predefined regions of interest (eg, vaping-related cues) will be collected and processed into fixation-related metrics such as fixation count, duration, and dwell time.
- HR and heart rate variability (HRV) will be monitored as indicators of autonomic arousal. HRV will be further processed into standard time-domain metrics, including the root mean square of successive differences, which reflects short-term parasympathetic activity, as well as frequency-domain metrics such as the low-frequency–to–high-frequency ratio, commonly interpreted as an index of sympathovagal balance [51].
- EDA will also be recorded to measure autonomic arousal and decomposed into tonic skin conductance levels and phasic SCRs using continuous decomposition analysis [52].

### Ethical Considerations

This study has been approved by the Yale University Human Subjects Committee (2000040783). The study protocol was prospectively registered on the Open Science Framework on November 26, 2025. Although not required for this study type, registration was completed to support transparency and reproducibility.

We have assessed that the study poses no greater than minimal risk to participants. The key ethical issues we considered include breaches of confidentiality and exposing individuals who have never used nicotine-containing e-cigarettes to vaping-related cues.

Under Connecticut law, the legal age to purchase e-cigarettes is 21 years, and it is illegal for anyone to sell, give, or deliver e-cigarettes to individuals below 21 years [53]. As our study recruits young adults aged 18 to 21 years and assesses past substance use behaviors, participants might have to disclose underage substance use behaviors. To ensure data privacy, participants will be assigned unique study identification numbers, and substance use behaviors and physiological data will be kept separate from identifying information. The consent form will clearly state these risks and list the protections available to participants. Participants will also be reminded that they can withdraw from the study at any time and have their records removed without penalty.

For the collected data, all information will be stored on secure Yale-managed encrypted servers accessible only to approved study personnel. Identifiable information will be stored separately from study data and linked through coded participant IDs. Physiological data collected in this study include eye-gaze direction, HR, and EDA. Eye tracking records only gaze direction toward predefined regions of interest and do not capture images, video, or pupil size. HR and EDA are collected as physiological signals that do not contain identifiable biological information. Study data will be retained for a minimum of 5 years following study completion in accordance with institutional policy. Deidentified, aggregate-level data may be shared for scientific purposes upon reasonable request and institutional review board approval.

We also considered the risk of exposing nonvapers to vaping-related information. Our youth advisory group shared that exposure to vaping cues is common in their environments, such as through peers, bus stops, and social media, even for those who do not vape. In a 6-week study on social media campaigns with teens (N=415), there was no reported evidence of increased initiation risks for nonusers exposed to e-cigarette educational messages [53,54]. As such, we assess that the risk of increasing participants’ susceptibility to initiate nicotine-containing vapes is low. At the end of the laboratory visits, we will also provide all participants with a resource sheet containing support services related to substance use and well-being.

### Analysis

#### Quantitative Analysis

All data will be screened for completeness and normality before analysis. Descriptive statistics will be computed for all subjective (eg, craving, sense of presence, social presence) and physiological (eg, HR, EDA, and eye-tracking) variables. Data preprocessing will include outlier detection, missing value treatment, signal quality checks, and feature extraction to ensure valid physiological readings.

##### Primary Analyses

For the primary aim of examining differences in psychological and physiological responses between vapers and nonvapers during VR cue exposure with and without social pressure, a mixed-model ANOVA will be conducted. Group (vapers vs nonvapers) will serve as the between-subjects factor, and cue condition (social pressure vs no social pressure) as the within-subjects factor. If the assumptions of normality or homogeneity of variance are violated, nonparametric alternatives such as the aligned rank transform ANOVA [55] will be used to preserve the ability to test interaction effects within the mixed design. Follow-up pairwise comparisons will be conducted with Bonferroni-adjusted *P* values.

##### Secondary Analyses

For the secondary objective, we will assess associations between multimodal physiological measures and subjective vaping-related outcomes. Correlation and regression analyses will first be conducted to examine pairwise relationships among eye-gaze metrics (eg, total fixations and mean fixation duration), HR, EDA, and subjective measures (eg, craving, dependence scores, and motivation to quit or susceptibility to initiate use). Depending on data distribution, the Pearson or Spearman correlations will be used. Partial correlations or multiple regression models will account for potential covariates such as age, gender, and other substance use.

To further explore multivariate relationships between physiological responses and vaping-related outcomes, exploratory regularized regression analyses will be conducted. Given the pilot sample size, we will use regularized logistic regression with the least absolute shrinkage and selection operator to examine whether aggregated physiological and subjective measures are associated with vaping status (vaper vs nonvaper). Candidate predictors will include summary features derived from eye-gaze behavior (eg, fixation count, proportion of fixation duration on vaping-related cues, and time to first fixation), HRV metrics derived from photoplethysmography signals (including time-domain indices such as root mean square of successive differences and frequency-domain measures such as low-frequency to high-frequency ratio), EDA (eg, mean number of SCRs, mean SCR amplitude, and mean skin conductance level), and relevant subjective responses (eg, craving, presence, and social presence).

Physiological signals will undergo standard preprocessing procedures, including signal quality checks, removal of segments affected by motion artifacts or signal instability, and aggregation into scene-level summary features. Participants will be excluded if more than 15% of their physiological data is missing, and EDA signals will be removed if more than 15% of values are zero or negative, indicating poor sensor contact [56,57]. The regularization parameter will be selected using cross-validation to reduce overfitting [58]. Given the exploratory nature of this analysis and the modest sample size, findings will be interpreted cautiously and used to identify candidate multimodal markers for future confirmatory studies rather than to develop generalizable prediction models.

### Qualitative Analysis

Qualitative data will be used to understand participants’ experiences in each VR cue scene and to contextualize the psychological and physiological data. Sessions will be transcribed using the Yale-managed instance of Zoom, a secure conferencing platform. Coding and analysis will be conducted via Dedoose (SocioCultural Research Consultants), using thematic analysis and a deductive approach [59]. Codes will first be generated from the data and then deductively by integrating codes informed by attention theory (eg, guided search model) [60] and the cognitive-affective personality system [61] to understand if and how the VR cues affect participants’ thoughts, emotions, and behaviors. We aim to reach saturation by interviewing all 40 participants.

## Results

The project received institutional review board approval in August 2025. We have completed 2 focus group discussions as part of our UCD process in September 2025. Development of the VR stimuli was completed in December 2025. Participant recruitment and data collection are currently ongoing. As of March 2026, data from 7 participants have been collected.

## Discussion

### Prospects

To our knowledge, this is the first experimental study to integrate multimodal psychophysiological data with immersive VR to understand nicotine vaping behaviors among young adults. The study is grounded in an interdisciplinary collaboration between psychologists, clinicians, VR developers, and designers, ensuring that both scientific rigor and user experience are prioritized. Through a co-design process with a youth advisory group, the VR scenes are iteratively refined to simulate everyday social contexts, dialogue, and environmental cues reflective of real-world vaping exposure.

By combining eye-tracking and physiological measures within a realistic, user-informed VR environment, this study moves beyond traditional self-report methods to capture implicit, real-time indicators of craving, attention, and arousal. Physiological data will also be integrated with demographic and personality variables to model vaping-related intentions and attitudes. This approach provides a novel pathway for identifying noninvasive digital biomarkers of nicotine use, addiction, and susceptibility. Together, these methodological innovations position this study as a foundation for creating scalable, adaptive tools for the detection and prevention of nicotine addiction and initiation risk; the framework could be readily extended to other addictive behaviors. The findings will help advance the development of real-time, psychophysiology-informed systems for substance use risk prediction and personalized intervention in youth populations.

### Limitations

This study protocol has several limitations. First, vaping initiation and cessation intentions will be assessed through self-report questionnaires at a single time point (at the time of the study) rather than via a longitudinal follow-up, which would allow validation of participants’ future vaping behaviors. The study is designed as an observational laboratory protocol and does not include follow-up, as it is not intended to influence or assess behavioral change over time. Accordingly, self-reported susceptibility and intention are established proxies for early indicators of nicotine use risk [42,62,63], and this study paradigm can inform future work validating VR-based biomarkers through longitudinal designs. Second, our VR stimuli focus primarily on visual immersion and do not incorporate multisensory elements such as olfactory cues, which are known to play an important role in e-cigarette use and craving [15]. Future work could expand to multisensory designs for greater ecological validity. Third, recruitment is limited to young adults aged 18‐21 years in the New Haven, Connecticut area, a sample likely to be predominantly university students and not fully representative of broader younger populations at risk. Fourth, one of the primary outcome measures, eye gaze direction, will be disclosed to participants before the VR session, which may influence natural viewing patterns. Additionally, both eye-tracking and physiological signals (HR and EDA) are susceptible to noise and motion artifacts [24], particularly in room-scale VR where participants move freely. We will mitigate these issues through standard preprocessing procedures and noise correction, but residual artifacts may still affect data quality. Despite these limitations, this study provides an important step in establishing the feasibility of VR-based psychophysiological assessment for nicotine use and risk prediction, laying the groundwork for larger-scale and more representative trials.

### Conclusions

This protocol describes a pilot study that integrates VR with multimodal psychophysiological assessment to examine vaping cue reactivity among young adults. By combining eye-tracking, HR, EDA, and self-report measures within bespoke VR environments, this study aims to establish the feasibility of VR-based biomarkers in distinguishing vaping status and modeling vaping-related intentions and susceptibility. The findings will guide the development and evaluation of scalable, noninvasive VR tools for identifying early markers of nicotine use risk. This work also lays the groundwork for extending and testing the approach with younger adolescents, with the long-term goal of supporting early detection and prevention of nicotine addiction and initiation.

## Abbreviations

EDA: electrodermal activity
HR: heart rate
HRV: heart rate variability
SCR: skin conductance response
USD: user-centered design
VR: virtual reality

## References

[1] Glantz S, Jeffers A, Winickoff JP (2022). Nicotine addiction and intensity of e-cigarette use by adolescents in the US, 2014 to 2021. JAMA Netw Open.

[2] Counotte DS, Spijker S, Van de Burgwal LH (2009). Long-lasting cognitive deficits resulting from adolescent nicotine exposure in rats. Neuropsychopharmacol.

[3] Goriounova NA, Mansvelder HD (2012). Short- and long-term consequences of nicotine exposure during adolescence for prefrontal cortex neuronal network function. Cold Spring Harb Perspect Med.

[4] Lu W, Aarsand R, Schotte K (2024). Tobacco and COPD: presenting the World Health Organization (WHO) Tobacco Knowledge Summary. Respir Res.

[5] O’Brien D, Long J, Quigley J, Lee C, McCarthy A, Kavanagh P (2021). Association between electronic cigarette use and tobacco cigarette smoking initiation in adolescents: a systematic review and meta-analysis. BMC Public Health.

[6] Meehan J, Heffron M, Avoy HM, Reynolds C, Kyne L, Cox DW (2024). The adverse effects of vaping in young people. Glob Pediatr.

[7] (2025). Key substance use and mental health indicators in the United States: results from the 2024 National Survey on Drug Use and Health. Substance Abuse and Mental Health Services Administration.

[8] Keijsers M, Vega-Corredor MC, Tomintz M, Hoermann S (2021). Virtual reality technology use in cigarette craving and smoking interventions (I “Virtually” Quit): systematic review. J Med Internet Res.

[9] Tatnell P, Atorkey P, Tzelepis F (2022). The effectiveness of virtual reality interventions on smoking, nutrition, alcohol, physical activity and/or obesity risk factors: a systematic review. Int J Environ Res Public Health.

[10] Weser VU, Duncan LR, Sands BE (2021). Evaluation of a virtual reality e-cigarette prevention game for adolescents. Addict Behav.

[11] Mazza M, Kammler-Sücker K, Leménager T, Kiefer F, Lenz B (2021). Virtual reality: A powerful technology to provide novel insight into treatment mechanisms of addiction. Transl Psychiatry.

[12] Pericot-Valverde I, Secades-Villa R, Gutiérrez-Maldonado J, García-Rodríguez O (2014). Effects of systematic cue exposure through virtual reality on cigarette craving. Nicotine Tob Res.

[13] Bordnick PS, Graap KM, Copp H, Brooks J, Ferrer M, Logue B (2004). Utilizing virtual reality to standardize nicotine craving research: a pilot study. Addict Behav.

[14] Paris MM, Carter BL, Traylor AC (2011). Cue reactivity in virtual reality: the role of context. Addict Behav.

[15] Traylor AC, Bordnick PS, Carter BL (2009). Using virtual reality to assess young adult smokers’ attention to cues. Cyberpsychol Behav.

[16] Winkler MH, Li Y, Pauli P, Mühlberger A (2023). Modulation of smoking cue reactivity by social context—Implications for exposure therapy in virtual reality. Front Virtual Real.

[17] Liu W, Andrade G, Schulze J, Doran N, Courtney KE (2022). Using virtual reality to induce and assess objective correlates of nicotine craving: paradigm development study. JMIR Serious Games.

[18] Schröder B, Kroczek A, Kroczek LOH, Ehlis AC, Batra A, Mühlberger A (2024). Cigarette craving in virtual reality cue exposure in abstainers and relapsed smokers. Sci Rep.

[19] Borrelli B, Weinstein D, Endrighi R (2025). Virtual reality for the prevention and cessation of nicotine vaping in youths: protocol for a randomized controlled trial. JMIR Res Protoc.

[20] Aneni K, Chen CH, Meyer J (2023). Identifying game-based digital biomarkers of cognitive risk for adolescent substance misuse: protocol for a proof-of-concept study. JMIR Res Protoc.

[21] Blackwell AKM, De-Loyde K, Brocklebank LA (2020). Tobacco and electronic cigarette cues for smoking and vaping: an online experimental study. BMC Res Notes.

[22] Upadhyaya HP, Drobes DJ, Thomas SE (2004). Reactivity to smoking cues in adolescent cigarette smokers. Addict Behav.

[23] Wei S, Bloemers D, Rovira A A preliminary study of the eye tracker in the Meta Quest Pro.

[24] Halbig A, Latoschik ME (2021). A systematic review of physiological measurements, factors, methods, and applications in virtual reality. Front Virtual Real.

[25] Schick SF, Blount BC, Jacob P (2017). Biomarkers of exposure to new and emerging tobacco delivery products. Am J Physiol Lung Cell Mol Physiol.

[26] Tamburin S, Dal Lago D, Armani F (2021). Smoking-related cue reactivity in a virtual reality setting: association between craving and EEG measures. Psychopharmacology (Berl).

[27] Kava CM, Soule EK, Seegmiller L (2021). “Taking up a new problem”: Context and determinants of pod-mod electronic cigarette use among college students. Qual Health Res.

[28] Schröder B, Mühlberger A (2022). Assessing the attentional bias of smokers in a virtual reality anti-saccade task using eye tracking. Biol Psychol.

[29] Singh KP, Camenga DR, Aneni K, Hieftje K, Wells JL (2025). A Clinical Lens on Pediatric Engineering.

[30] Halcomb EJ, Davidson PM (2006). Is verbatim transcription of interview data always necessary?. Appl Nurs Res.

[31] Courtney KE, Liu W, Andrade G, Schulze J, Doran N (2024). Attentional bias, pupillometry, and spontaneous blink rate: eye characteristic assessment within a translatable nicotine cue virtual reality paradigm. JMIR Serious Games.

[32] Wei S, Freeman D, Harris V, Rovira A (2024). A randomised controlled test in virtual reality of the effects on paranoid thoughts of virtual humans’ facial animation and expression. Sci Rep.

[33] Wei S, Freeman D, Rovira A (2025). Virtual humans in virtual reality mental health research: systematic review. JMIR XR Spatial Comput.

[34] Wei S, Freeman D, Rovira A Visual attention and virtual human facial animations in virtual reality (VR): an eye-tracking study.

[35] Shimmer3R GSR+ unit. Shimmer.

[36] Ronca V, Martinez-Levy AC, Vozzi A (2023). Wearable technologies for electrodermal and cardiac activity measurements: a comparison between Fitbit Sense, Empatica E4 and Shimmer GSR3. Sensors (Basel).

[37] Gupta K, Zhang Y, Gunasekaran TS, Krishna N, Pai YS, Billinghurst M (2024). CAEVR: Biosignals-driven context-aware empathy in virtual reality. IEEE Trans Visual Comput Graphics.

[38] Nechyporenko A, Frohme M, Strelchuk Y (2024). Galvanic skin response and photoplethysmography for stress recognition using machine learning and wearable sensors. Appl Sci.

[39] Fucito LM, Ash GI, Wu R (2025). Wearable intervention for alcohol use risk and sleep in young adults: a randomized clinical trial. JAMA Netw Open.

[40] Kotz D, Brown J, West R (2013). Predictive validity of the Motivation to Stop Scale (MTSS): a single-item measure of motivation to stop smoking. Drug Alcohol Depend.

[41] Foulds J, Veldheer S, Yingst J (2015). Development of a questionnaire for assessing dependence on electronic cigarettes among a large sample of ex-smoking E-cigarette users. Nicotine Tob Res.

[42] Pierce JP, Choi WS, Gilpin EA, Farkas AJ, Merritt RK (1996). Validation of susceptibility as a predictor of which adolescents take up smoking in the United States. Health Psychol.

[43] Sontag JM, Wackowski OA, Hammond D (2019). Baseline assessment of noticing e-cigarette health warnings among youth and young adults in the United States, Canada and England, and associations with harm perceptions, nicotine awareness and warning recall. Prev Med Rep.

[44] Mittal A, Du A, Merz W, Myers MG, Crotty Alexander LE, Doran N (2022). Impulsivity-related personality traits as predictors of e-cigarette use among young adults over time. Subst Use Misuse.

[45] Cyders MA, Littlefield AK, Coffey S, Karyadi KA (2014). Examination of a short English version of the UPPS-P Impulsive Behavior Scale. Addict Behav.

[46] Victor SE, Klonsky ED (2016). Validation of a brief version of the Difficulties in Emotion Regulation Scale (DERS-18) in five samples. J Psychopathol Behav Assess.

[47] Eidenmueller K, Hoffmann S, Kammler-Sücker K (2025). Reactivity to smoking cues in a social context: virtual reality experiment. JMIR Form Res.

[48] Harms C, Biocca F Internal consistency and reliability of the networked minds measure of social presence. https://web-archive.southampton.ac.uk/cogprints.org/7026/1/Harms_04_reliability_validity_social_presence_(Biocca).pdf.

[49] Witmer BG, Singer MJ (1998). Measuring presence in virtual environments: a presence questionnaire. Presence.

[50] Kim YM, Rhiu I (2024). Development of a Virtual Reality System Usability Questionnaire (VRSUQ). Appl Ergon.

[51] Eckberg DL (1997). Sympathovagal balance: A critical appraisal. Circulation.

[52] Benedek M, Kaernbach C (2010). A continuous measure of phasic electrodermal activity. J Neurosci Methods.

[53] Dube N (2023). Connecticut’s e-cigarette laws. https://www.cga.ct.gov/2023/rpt/pdf/2023-R-0098.pdf.

[54] England KJ, Edwards AL, Paulson AC, Libby EP, Harrell PT, Mondejar KA (2021). Rethink Vape: development and evaluation of a risk communication campaign to prevent youth E-cigarette use. Addict Behav.

[55] Wobbrock JO, Findlater L, Gergle D, Higgins JJ (2011). The aligned rank transform for nonparametric factorial analyses using only ANOVA procedures.

[56] Wei S, El Ali A, Cesar P, Freeman D, Rovira A Physiological responses to affective virtual coach design in a VR fear of heights consultation.

[57] Taylor S, Jaques N, Fedor S, Sano A, Picard R Automatic identification of artifacts in electrodermal activity data.

[58] Zou H, Hastie T (2005). Regularization and variable selection via the Elastic Net. J R Stat Soc Series B Stat Methodol.

[59] Braun V, Clarke V (2006). Using thematic analysis in psychology. Qual Res Psychol.

[60] Wolfe JM (2021). Guided Search 6.0: An updated model of visual search. Psychon Bull Rev.

[61] Mischel W, Shoda Y (1995). A cognitive-affective system theory of personality: reconceptualizing situations, dispositions, dynamics, and invariance in personality structure. Psychol Rev.

[62] Cole AG, Kennedy RD, Chaurasia A, Leatherdale ST (2019). Exploring the predictive validity of the susceptibility to smoking construct for tobacco cigarettes, alternative tobacco products, and e-cigarettes. Nicotine Tob Res.

[63] Seo DC, Kwon E, Lee S, Seo J (2020). Using susceptibility measures to prospectively predict ever use of electronic cigarettes among adolescents. Prev Med.

